# The influence and impact of livelihood capitals on livelihood diversification strategies in developing countries: a systematic literature review

**DOI:** 10.1007/s11356-023-27638-2

**Published:** 2023-05-17

**Authors:** Nusrat Habib, Anoma Ariyawardana, Ammar Abdul Aziz

**Affiliations:** School of Agriculture and Food Sciences, The University of Queensland, Brisbane, 4072 Australia

**Keywords:** Livelihood capitals, Livelihood diversification, Poverty reduction, Systematic review, Developing countries

## Abstract

Livelihood diversification is an essential strategy for managing economic and environmental shocks and reducing rural poverty in developing countries. This article presents a comprehensive two-part literature review on livelihood capital and livelihood diversification strategies. Firstly, it identifies the role of livelihood capital in determining livelihood diversification strategies, and secondly, it assesses the role of livelihood diversification strategies in reducing rural poverty in developing countries. Evidence suggests that human, natural, and financial capitals are the primary determining assets of livelihood diversification strategies. However, the role of social and physical capital with livelihood diversification has not widely been studied. Education, farming experience, family size, land holding size, access to formal credit, access to market, and membership in village organizations were the major influencing factors in the adoption process of livelihood diversification strategies. The contribution of livelihood diversification in poverty reduction (SDG-1) was realized through improved food security and nutrition, increased income level, sustainability of crop production, and mitigating climatic vulnerabilities. This study suggests enhanced livelihood diversification through improved access to and availability of livelihood assets is vital in reducing rural poverty in developing countries.

## Introduction

Smallholder farmers in rural areas of developing countries represent over two-thirds of the global poor and food-insecure population (FAO et al. 2014). These rural economies of developing countries are characterized by high dependence on agriculture which is prone to shocks such as weather and natural disasters (Bezabih et al. 2014), financial risks (Reddy 2015), price and production risks (Meuwissen et al.^2015^), and policy risks (Akcoaz and Oskan 2005). In the event of a shock to the agricultural sector, livelihoods of agriculture-dependent rural communities are severely affected (Abid et al. 2016; Imran et al. 2018). Arguably, an effective way to reduce livelihood risks and rural poverty in developing regions is by adopting diversified livelihood strategies (Lemi 2009).

According to Ellis (2000), livelihood includes the assets (human, natural, social, physical, and financial capitals), the activities, and the access to these activities (intermediated by institutes and social interactions) necessary for a means of living. Similarly, Bryceson (2002) also described livelihoods as strategies people adopt to satisfy their needs and earn a living. Its primary purpose is to earn an income and sustain a better life (Gwimbi 2009; Mutopo 2014). Livelihood can be considered sustainable when it is sufficient to prevent poverty and expand the overall wellbeing of an individual or a household (FAO 2013).

Rural livelihoods are the systems of rural communities that get a standard of living, whether their livelihoods are secure or at risk over time. Livelihood insurance is to ensure availability, accessibility, and possession of reserves, assets, and resources to cope with shocks to go through eventualities and counteract risk (Barrett et al. 2001; Gladwin et al. 2001). The process by which rural families build a varied range of activities and resources to endure and expand their living standards is characterized as “livelihood diversification” (Ellis 2000).

Livelihood diversification is a crucial approach for poverty reduction for rural households in different parts of the developing world (Assan 2014; Ellis 2000). It is aimed at securing improved livelihood standards by decreasing risk exposure and poverty, expanding income, improving security, and expanding wealth (Yaro 2006). A study conducted by Food and Agriculture Organization (FAO) on poverty and farming systems found livelihood diversification to play an essential role in managing livelihood risks and poverty reduction in South Asia (FAO 2015; WB 2007).

In order to adopt livelihood diversification strategies to obtain better living standards, rural households have to be able to make cash, build resources, and spread their sources of income with a combination of farm and non-farm activities (Ellis and Freeman 2004). Though farming is prevalent in many rural areas, livelihoods are intricate. Rural households often maintain a diversified portfolio of interests, among which crops and livestock productions appear along with many other contributions to household livelihoods (Barrett et al. 2005; Smith 2004). Poor smallholders devoid of the required resources often pursue alternate income by engaging in lower pay back and from time to time risky non-farm activities to compensate for any losses incurred during agricultural production and distribution (Barrett et al. 2001). On the other hand, the increment in income and accumulation of wealth is the primary motivation for diversification of the income stream among the more affluent households (Haggblade et al. 2007).

Many empirical studies have reported on the dynamics of livelihood capital, income, rural poverty, and livelihood diversification. These studies have shown that livelihood capitals are critical in determining livelihood diversification (Ansoms and McKay 2010; Iiyama et al. 2008; Mutenje et al. 2010; Shanta et al. 2018). To achieve a positive livelihood diversification outcome, individuals need to possess different livelihood capitals in hand (Iiyama et al. 2008). The choice of livelihood diversification also increases with livelihood capital possession (Mutenje et al. 2010).

The United Nations (UN) 2030 agenda, which includes the 17 Sustainable Development Goals (SDGs), was intended to protect the planet, increase prosperity, and improve the standard of living and lives of people (UN 2016). Given many goals, the dynamic interactions between the SDGs are inevitable; however, our understanding of these interactions remains limited (Allen et al. 2018). Correlations between SDGs mostly point towards synergies and indicate trade-offs (Pradhan et al. 2017). For some SDGs, these interactions are straightforward, while others are relatively unknown (Pradhan et al. 2017). Given the significance of these goals, policy-makers must obtain timely and relevant knowledge to enable prospective alleviation or adjustment guidelines on SDG trade-offs. Therefore, this study will assess the impact of livelihood diversification in reducing poverty (related to SDG-1 “no poverty”) in relation to the other associated SDGs (Goal 2: zero hunger, Goal 5: gender equality, Goal 8: decent work and economic growth, Goal 10: reduce inequalities, Goal 12: sustainable consumption/Goal 12: sustainable production, and Goal 13: climate action). The selected combination of SDGs was based on the UN definition for poverty within the sustainable development plan, which reflects poverty as the absence of crucial services such as gender equality, hunger, social discrimination and segregation, and lack of involvement in decision-making (UN 2015).

Although there is an abundance of research on the relationship between livelihood capitals and livelihood diversification (Ansoms and McKay 2010; Iiyama et al. 2008; Mutenje et al. 2010; Shanta et al. 2018), the impact and link between livelihood diversification and poverty reduction have rarely been investigated. Additionally, there are very few review studies on rural livelihood diversification patterns in a developing country context (Barrett et al. 2001; Oduniyi and Tekana 2019; Sarah 2019). This study is aimed at filling that research gap. The study’s objective is to systematically review relevant literature to address two research questions: (1) How does livelihood capital influence smallholder livelihood diversification strategies in developing countries? (2) What are the contributions of livelihood diversification in reducing poverty among smallholders in developing countries? This review will help identify research gaps and future research opportunities, and it could inform policymakers and potentially enhance the development of future livelihood diversification strategies.

## Review methods

The literature review of this study adopted the review method in line with the Campbell (2014) guidelines for systematic review in social science, which demand that all steps in the review are documented and made transparent.

### Scope

To be considered for inclusion in the review, the selected studies had to be on diversification and its impact on the livelihood of smallholders with a particular focus on low-income developing countries. The diversification strategies can include on-farm or off-farm activities, but the main selection criterion for this study was the livelihoods of the farming community. As the focus of this study is to analyze the impact of diversification on the selected SDGs, we only considered studies published between January 2000, which was when the Millennium Development Goals (MDGs) were established, and December 2021. The MDGs were then renamed Sustainable Developmental Goals (SDGs) in 2015 (UN 2015). The eligible studies had to report on the impact of at least one of the following indicators of livelihood capitals: human, social, physical, natural, and financial. Only the publications in English were included in the study.

### Search

We used three major social science databases: Science Direct, Web of Science, and ABI/INFORM Collection to conduct our literature review. Brocke et al. (2009) advised that a systematic review literature should be initiated with an extensive formation of what is known about the topic. To do this, the study initiated a random Google search to identify relevant keywords (search terms). The results of this search were then used to develop a matrix of keywords, which was then refined and applied in the selected databases.

The initial search was carried out using the keywords “livelihood diversification” AND “rural diversification” AND “agricultural diversification” AND “poverty reduction, as well as “livelihood capitals” AND “livelihood diversification” AND “developing countries.” The other search terms such as “livelihood,” “livelihoods,” “diversification,” “least developed countries,” and “low developed countries” were included to identify other relevant literature. Furthermore, after thoroughly reading all the relevant articles, the references of these articles were cross-checked to identify other possible studies. We continued to conduct regular searches on Google Scholar throughout the review to ensure newly published within the scope articles were included.

### Screening

**Fig. 1:**
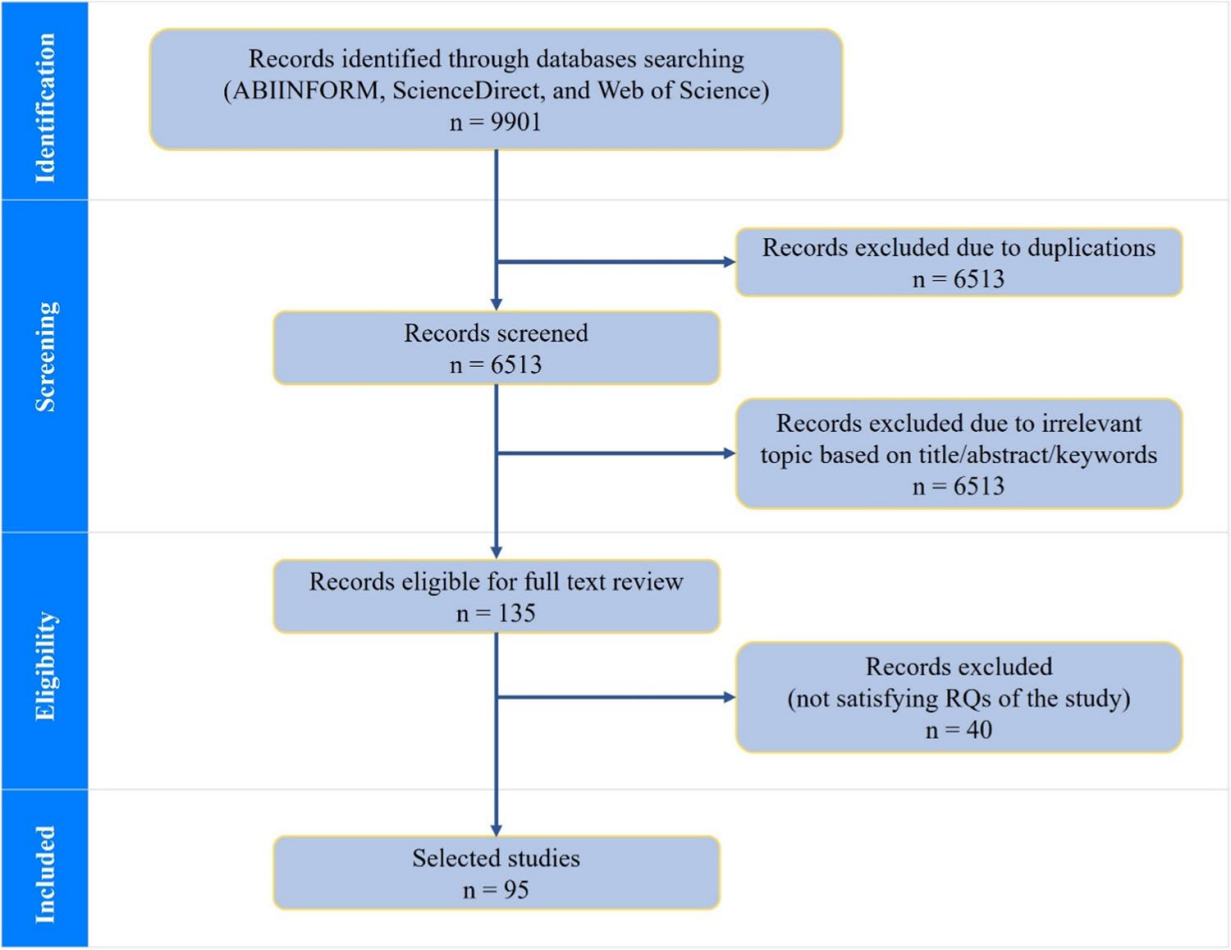
Flow diagram of included studies following PRISMA framework

The initial number of articles identified were 9901, and these were reviewed to ensure it met the inclusion criteria. An article would be added to the final list of the review if: (1) it addressed the livelihood diversification and examined the impact of diversification on livelihoods of smallholders and poverty reduction; and (2) the origin of the selected article must be in one of the developing countries. The selected research articles were further screened by reading the titles, abstracts, and keywords, resulting in the exclusion of 3253 irrelevant articles. The remaining research articles were then pooled and filtered to remove any duplication in Endnote. This resulted in a total of 135 research articles. A full-text reading screening was conducted, and the process excluded a further 40 articles and left behind 95 articles in the selection list, which were included in this review for data extraction and synthesis. Figure 1 provides an overview of the selection process.

### Data extraction and critical appraisal

The data was extracted and analyzed to specifically address the research questions posed in this review article. The extracted data include details on the authorship, place and date of publication, study aim(s), country of study, the method adopted, and type and target population of intervention in the selected studies. We extracted information which were primarily focused on the relationship between diversification and livelihoods.

### Synthesis

The main aim of any systematic review is to synthesize the information obtained in the data extraction process. We were especially interested in the benefits of diversification on smallholder livelihoods in developing countries. We synthesized the information from the perspective of food security and nutrition, poverty reduction, improvement in income, and betterment in social and physical indicators. We also focused on the impact of key livelihood capitals (social, physical, human, financial, or natural) on livelihood diversification.

## Results and discussion

### Descriptive analysis

**Fig. 2:**
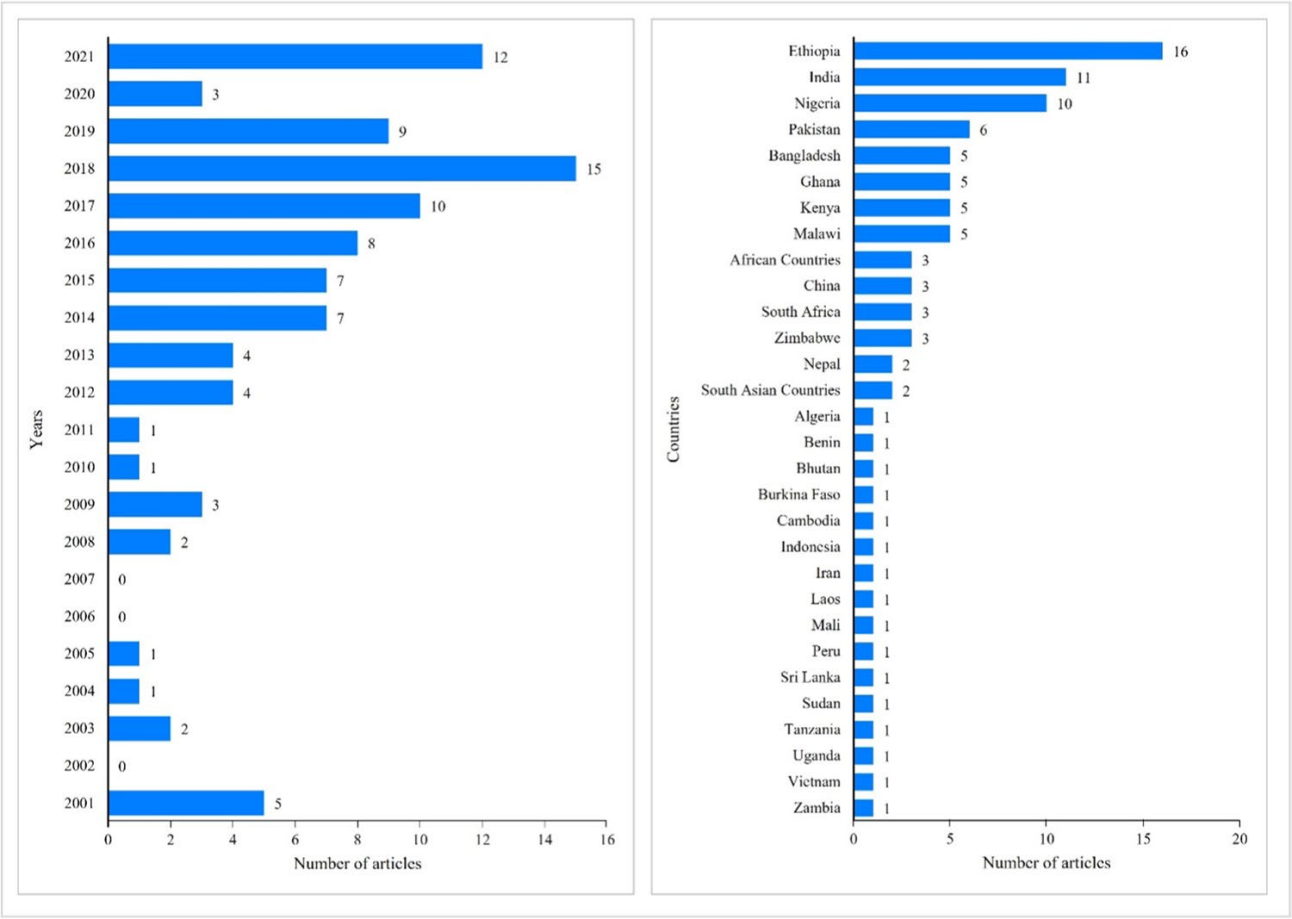
Year and country-wise distribution of selected studies

The 95 studies included in the synthesis of the systematic review were published between January 2000 and December 2021. There were 15 studies published in 2018, the highest number of publications on livelihood diversification strategies in a single year. The geographic locations identified in the studies were widely spread across the developing countries; 64.2% of the total studies were located in Africa while 35.8% of studies were located in Asia. The studies were conducted in 30 different countries. Ethiopia hosted the most significant number of studies (16), followed by India (11) and Nigeria (10). A summary of the publication year and geographic location of the selected studies is presented in Fig. 2.

The selected studies were drawn from two primary publication sources: (1) journal articles (97.5 %) and (2) research thesis (2.5%). The overall scope of the studies could be grouped into four themes: (1) agricultural diversification, which involves a range of agricultural activities (crop varieties and species, or animal breeds, to farms or farming communities) and also includes the change in cropping pattern and transformation of workforce from agriculture work to other associated activities like poultry, livestock, fisheries; (2) crop diversification, which involves a shift from single cropping system to multi-cropping systems; (3) income diversification—defined as the process of switching from low-value crop to high-value crop production, or increasing the number of income sources; and (4) livelihood diversification—a strategy which can include different forms of diversification such as agricultural, crop, and income diversification. A total number of 38 studies focused on livelihood diversification. Twenty-three studies looked at agricultural diversification, 14 on income diversification, 13 on crops, and 45 on overall livelihood diversification.

**Fig. 3:**
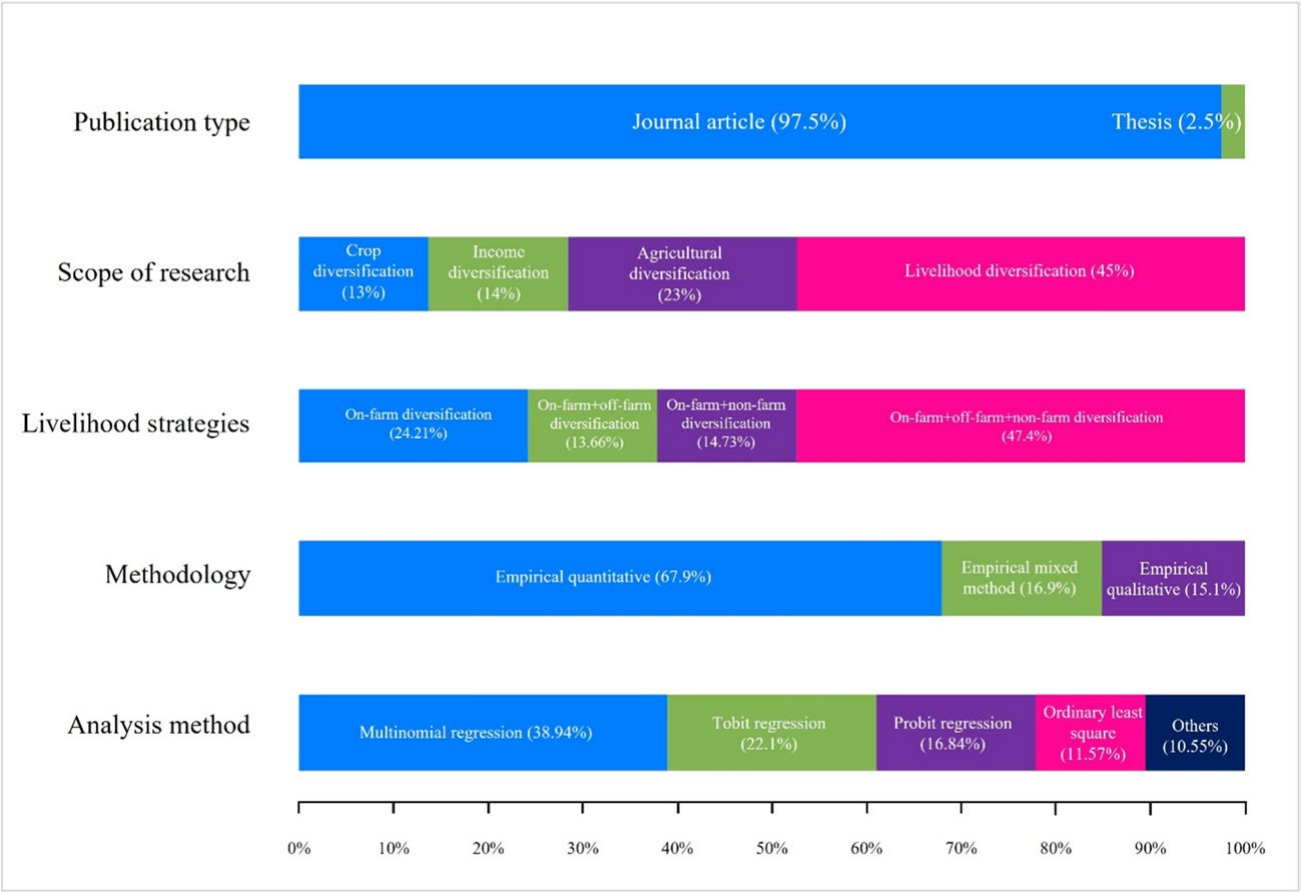
Overview of selected studies

Findings revealed that most of the households in the studies adopted diversification with a combination of on-farm, off-farm, and non-farm strategies (44.4%). On the other hand, 24.21%, 13.66%, and 14.73% of the sample studies represented that the sample households were able to diversify their strategies into on-farm only, on-farm + off-farm, and on-farm + non-farm, respectively. In the 95 selected studies, 67.9% were primarily quantitative compared to 15.1% for qualitative research, and 16.9% adopted a mixed-methods approach. This indicated a preference for quantitative methodologies in research on livelihood diversification strategies. In the analyses of the selected studies, diverse analytical techniques were adopted, and the most common methods were probit, logit, tobit regression models, ordinary least square model, and two-stage least square method (Fig. 3).

### Factors of livelihood capitals influencing the diversification process

This section reviews the influence of the five capitals—human, physical, natural, financial, and social—on the diversification process of livelihoods. The synthesis revealed that human capital was the most discussed asset class by more than half of the selected studies (76.84%). The other financial, natural, physical, and social assets were discussed by 65.26%, 64.21%, 49.47%, and 35.78%, respectively. Table 1 presents a summary of the identified livelihood themes and sub-themes.

**Table 1:** Identified themes and sub-themes

Themes	Sub-themes	No. of articles discussing themes
Human capital	Age, education, access to trainings, farming experience, family size, and gender of household head (dummy)	73 (76.84)
Financial capital	Access to formal or informal credit, remittances, savings, and non-farm income sources	62 (65.26)
Natural capital	Farmland holding size, livestock inventory, and climatic variability	61 (64.21)
Physical capital	Access to road/market, road infrastructure, storage facility, and farm- or household-used machinery	47 (49.47)
Social capital	Membership in any village organization, social status of household head, and leadership role	34 (35.78)

in parentheses are percentage

#### Human capital

Human capital is perhaps the most critical asset because its core value is essential in ensuring the ability to use the other four capitals. Human capital refers to “the knowledge, skills, creativity, good health, capability to labor, and education level that all together enable people to perform diverse livelihood strategies in achieving their livelihood goals” (Bealu 2019; DFID 1999). According to Martin and Lorenzen (2016), although households share related physical (regarding access to regional markets, etc.) and agroecological (regarding climate change perspective) conditions, socio-economic factors play a crucial role in differentiating livelihood diversification strategies within the household. These factors include family size, age, dependency ratio, access and level of education, access and availability of land, access to assets, and irrigation facilities (Dilruba and Roy 2012). In the selected studies, the critical socio-economic factors that impact livelihood diversification strategies identified include the level of education, access to training, farming experience or age of the decision-maker, and the family size (Abeje et al. 2019; Makate et al. 2016; Monika et al. 2017). Only 12 studies in the selected list considered the gender of household head as an important factor in the livelihood diversification process.

In a study on income diversification in Indonesia, Schwarze and Zeller (2005) found that access to education and training services were the two critical factors in human capital that influence diversification strategies. These two factors can improve employment opportunities in the non-farm sector, a potential income diversification strategy (Adjimoti and Kwadzo 2018). Gautam and Andersen (2016) also determined that education was the most influencing indicator in the livelihood diversification process. Meena (2018) described human capital as the main asset in generating livelihood earnings in developing countries. However, many human capital and gender-disaggregated data show that developing countries have primarily unskilled human capital (Awudu and Anna 2001; Sarah 2019). Women are also more vulnerable in terms of human development indicators as they have less access to education and other basic facilities (Sadia and Farah 2017).

Shanta et al. (2018) found that an increase in age and farming experience positively impacts livelihood diversification strategies. However, this contrasts with other studies (Akaakohol and Aye 2014; Bealu 2019; Gebru et al. 2018; Onunka and Olumba 2017), demonstrating that an increase in age and farming experience negatively influenced diversification decisions. A possible explanation for the contradictory findings may be related to the fact that as the age of the farm household increases, the capability of diversifying livelihood activities decreases. Aging farmers are more likely to converge on on-farm activities to maintain their subsistence consumption needs (Abimbola 2013; Bealu 2019). Oduniyi and Tekana (2019) in South Africa found that younger farmers were more interested in adopting livelihood diversification than their old age counterparts. Table 2 summarizes the key factors of human capital influencing diversification.

**Table 2:** Summary of key findings of human capital reported in reviewed studies

Main finding	Factors	Influence on diversification	Reference
Factors like family size, age, gender of the household head, education, and experience in farming can affect the household decision-making manner in adopting livelihood diversification strategies.	Education	+	Abimbola (2013); Adjimoti and Kwadzo (2018); U. I. Ahmed et al. (2017); Akaakohol and Aye (2014); Anjani et al. (2012); Dilruba and Roy (2012); Dympep et al. (2018); Gautam and Andersen (2016); Gebru et al. (2018); Gururaj et al. (2017); Jiao et al. (2017); Mango et al. (2014); Oduniyi and Tekana (2019); Schwarze and Zeller (2005); Shakila et al. (2019)
		-	Benmehaia and Brabez (2016); Monika et al. (2017); Shanta et al. (2018)
	Family size	+	Abeje et al. (2019); Abimbola (2013); Anjani et al. (2012); Cynthia (2018); Dilruba and Roy (2012); Gautam and Andersen (2016); Jones et al. (2014); Monika et al. (2017); Oduniyi and Tekana (2019); Raphael and Matin (2009); Alemayehu et al. (2021)
		-	Akaakohol and Aye (2014); Bealu (2019); Kebede et al. (2014);
	Age	+	Abeje et al. (2019); Adjimoti and Kwadzo (2018); Dilruba and Roy (2012); Dympep et al. (2018); Gautam and Andersen (2016); Gururaj et al. (2017); Raphael and Matin (2009); Shanta et al. (2018); Yuya and Daba (2018)
		-	Abimbola (2013); Anjani et al. (2012); Bealu (2019); Benmehaia and Brabez (2016); Cynthia (2018); Gebru et al. (2018); (Jiao et al. 2017); Jones et al. (2014); Monika et al. (2017); Oduniyi and Tekana (2019); Alemayehu et al. (2021)
	Farming experience	+	Gautam and Andersen (2016); Gururaj et al. (2017); Kebede et al. (2014); Monika et al. (2017); Shakila et al. (2019); Shanta et al. (2018)
		-	Akaakohol and Aye (2014); Benmehaia and Brabez (2016); Oduniyi and Tekana (2019)
	Gender of household head	+	Abeje et al. (2019); Abimbola (2013); Adjimoti and Kwadzo (2018); Bealu (2019); Oduniyi and Tekana (2019); Raphael and Matin (2009)
		-	Cynthia (2018); Gautam and Andersen (2016); Huang et al. (2014); Jones et al. (2014); Monika et al. (2017); Shanta et al. (2018); Alemayehu et al. (2021)

#### Financial capital

Financial capital, such as savings, cash flows, and credit-providing organizations, describes to the different financial resources used by people to attain their livelihood objectives (DFID 1999). The primary sources of financial capital identified in the selected studies were access to formal credit facilities and family income with a combination of savings, off-farm income, on-farm income, and remittance. The on-farm income was observed to be the primary income source for smallholders’ livelihood in developing countries (Israr et al. 2017; Makate et al. 2016; Mango et al. 2014; Njeru 2013). Excepting earned income, the most general types of cash inflows were pensions, or other transfers from the state (Gebru et al. 2018).

When considering the research synthesis of this study, we measured financial capital from two aspects: the accessibility to formal credit and savings. Increased access to formal credit provides households with an enhanced ability to diversify their income stream and improve their livelihood (Abeje et al. 2019). Some studies have shown that households with access to formal credit will increase livelihood diversification, such as purchasing advanced technology or investing in small businesses (Akaakohol and Aye 2014; Kanwal et al. 2016; Shakila et al. 2019). Contrary to this, other studies have found that even with access to formal credit, smallholder households could not diversify their livelihood strategies by getting involved in other income-generating activities apart from farming (Oduniyi and Tekana 2019; Raphael and Matin 2009). Similarly, Sarah (2019) study concluded that formal credit access has generally increased access to agricultural input only to promote agricultural intensification rather than diversifying their livelihoods from the farming sector in the African developing countries. While women of developing countries with high percentage do not have access to financial resources such as women can not acquire monetary benefit in terms of farm land lease or loan from banks (Ecker 2018). Most of the formal credits have limitation to provide land ownership proof and in developing countries women have almost no land ownership status. Limited access to natural resources for women becomes a reason to have no access to formal loan from many banks in developing countries (Miltone 2015).

Increased savings can strengthen people’s risk-bearing capacity enabling households to change their livelihood diversification strategy in the time of any natural disaster to maintain usual living standards (Abeje et al. 2019; Bealu 2019; Gebru et al. 2018). This also indicates that natural disasters are a push factor for the adoption of livelihood diversification. For example, Benmehaia and Brabez (2016) and Jiao et al. (2017) found that despite households having higher family savings, they were still reluctant to adopt any form of livelihood diversification strategies and would only do so when they were affected by natural disasters. This implies that households with only higher financial capital accessibility do not assure the adoption of livelihood diversification as other livelihood assets (human, natural, physical, and social) contribute to the livelihood diversification process in developing countries. A summary of the key factors of financial capital influencing diversification is presented in Table 3.

**Table 3:** Summary of key findings of financial capital reported in reviewed studies

Main findings	Factors	Influence on diversification	Reference
Non-farm income has become a significant factor in livelihood diversification process. Households who have better access to credit are more likely to participate in livelihood diversification than their counterparts.	Access to credit or banks	+	Abeje et al. (2019); Adjimoti and Kwadzo (2018); Akaakohol and Aye (2014); Anjani et al. (2012); Bealu (2019); Gautam and Andersen (2016); Kanwal et al. (2016); Shakila et al. (2019); Shanta et al. (2018); Alemayehu et al. (2021)
		-	Gebru et al. (2018); Oduniyi and Tekana (2019); Raphael and Matin (2009);
	Family income such as savings and remittance	+	Abeje et al. (2019); Abimbola (2013); Bealu (2019); Dilruba and Roy (2012); Dympep et al. (2018); Gebru et al. (2018)
		-	Benmehaia and Brabez (2016); Jiao et al. (2017); Shanta et al. (2018)

#### Natural capital

Natural capital plays a crucial role in rural areas, where majority of the rural people engaged in some type of farming activities. It is not only important for livelihood creation, but it is also significant to sustain life itself. The range of natural resources might involve elusive public goods such as climate change, to assets such as tree, land and water, applied directly for production (DFID 1999). There is vast literature analyzing the impact of land size on livelihood diversification. A review study undertaken by Harris (2014) concluded that bigger farm size was an essential factor that influence smallholder farmers to adopt crop diversification. It has been revealed that in Zimbabwe, an increase of land size by one acre would increase the probability of adoption of crop diversification by 15.8% (Makate et al. 2016). In Nigeria, Asfaw et al. (2018) also found that farm size had a significant and positive impact on adopting diversified livelihood strategies. Similar positive relationships were also determined by Adjimoti and Kwadzo (2018) in Benin, Bealu (2019) in Ethiopia, Kanwal et al. (2016) in Pakistan, Kebede et al. (2014) in Ethiopia, Monika et al. (2017) in India, and Shakila et al. (2019) in Bangladesh. Contrary to this, Abeje et al. (2019) found that more extensive land holding was associated with lower diversification in Ethiopia, mainly because large farm size holders specialized in a specific cropping system. Birthal et al. (2015) considered large-scale farmers better equipped to deal with risks associated with traditional production systems due to their high value.

Climate change represents a substantial threat to existing agricultural production system. It poses severe challenges to millions of poor farmers who live in areas often located in the developing regions’ arid or semi-arid zones (Huang et al. 2014). Recent studies have demonstrated a positive association between livelihood diversification and climate change. Climate variability has resulted in more farmers adopting livelihood diversification strategies to minimize the impacts of climatic shocks on smallholder production systems (Anjani et al. 2012; Birthal et al. 2015; Njeru 2013). Only Yuya and Daba (2018) mentioned that climate variability adversely affects the adoption of a diversified livelihood system for smallholder farmers in China. Makate et al. (2016) concluded that more effective implementation of diversified cropping systems decreased vulnerability to climate change and adaptability in smallholder farming systems in southern Africa by significantly improving their crop yields, income, food security, and nutrition. A summary of the key factors of natural capital influencing diversification is presented in Table 4.

**Table 4:** Summary of key findings of natural capital reported in reviewed studies

Main findings	Factors	Influence on diversification	Reference
The small and medium landholding households are more likely to diversify their livelihoods than functionally landless and significant landholding households.	Land holding size	+	Adjimoti and Kwadzo (2018); Bealu (2019); Kanwal et al. (2016); Kebede et al. (2014); Monika et al. (2017); Shakila et al. (2019)
		-	Abeje et al. (2019); Anjani et al. (2012); Benmehaia and Brabez (2016); Cynthia (2018); Dympep et al. (2018); Gebru et al. (2018); Schwarze and Zeller (2005)
	Climate variability	+	Birthal et al. (2015) Anjani et al. (2012); Dilruba and Roy (2012); Gautam and Andersen (2016); (Huang et al. 2014); Martin and Lorenzen (2016); Miltone (2015); Njeru (2013); Tanvir et al. (2015)
		-	Yuya and Daba (2018)

#### Physical capital

Physical capital includes private and public infrastructure, goods, and services required to maintain livelihoods. Public infrastructures such as water supply, roads, hospitals, schools, sanitation, energy, and access to information help people meet their basic needs and be more productive. Safe shelter and equipment required to sustain livelihoods are also vital, and for farmers, this might contain farming tools and livestock (DFID 1999). Previous studies have shown that poor infrastructure can reduce access to water supplies and energy, inhibiting income generation activities. For farmers, machinery and infrastructure are required to transport fertilizer, produce, and access markets. The synthesis of the selected studies illustrated that access to roads/markets and access to machinery are the main physical assets driving livelihood diversification strategies (Adjimoti and Kwadzo 2018; Birthal et al. 2015; Dilruba and Roy 2012). Makate et al. (2016) clearly stated that the main factors enabling households to access more lucrative strategies are physical assets and access to infrastructure.

In India, Anjani et al. (2012) found that farmers who lived closer to roads were more likely to participate in markets and grow a higher diversity of crop mix than farmers living in remote areas. Birthal et al. (2015) assumed that the extent of paved roads was positively linked to the adoption of diversified livelihood strategies that include livestock diversification (dairy, fisheries, and poultry). Shanta et al. (2018) concluded in their study that the major constraints for adopting diversified livelihood strategies by smallholders in rural areas in Nepal were poor transportation facilities and connections to the markets. A summary of the key factors of physical capital influencing diversification is presented in Table 5.

**Table 5:** Summary of key findings of physical capital reported in reviewed studies

Main findings	Factors	Influence on diversification	Reference
Overall, the location where the respondent is residing and land holding size has a positive and significant influence on participation livelihood diversification.	Access to roads	+	Anjani et al. (2012); Birthal et al. (2015)
		-	Jiao et al. (2017); Schwarze and Zeller (2005)
	Access to market	+	Monika et al. (2017); Raphael and Matin (2009); Shanta et al. (2018)
		-	Abeje et al. (2019); Akaakohol and Aye (2014); Dilruba and Roy (2012); Gebru et al. (2018); Oduniyi and Tekana (2019); Shanta et al. (2018); Alemayehu et al. (2021)
	Agricultural machinery	+	Benmehaia and Brabez (2016); Birthal et al. (2015)

#### Social capital

All social relationships are considered social capitals (Scoones 2009). In a broader sense, social capital emphasizes the value of networks, membership in more formalized groups of society, relationships of trust, and reciprocal interactions which people draw in pursuit of their livelihood objectives (DFID (1999). The review indicates that a cooperative member has a higher probability of participating in livelihood diversification strategies. Shanta et al. (2018) found becoming a member of any developmental group or organization can increase the chances of livelihood diversification. Many studies show that in times of economic vulnerabilities, smallholders use their resources to improve the livelihoods of their households. Studies have shown that smallholder farmers have joined labor organizations at the village level to take collective decisions to gain maximum benefit for the group members (Makate et al. 2016; Mango et al. 2018).

Interestingly, improved access to agricultural extension offices was found to affect livelihood diversification strategies negatively. This may be because farmers having improved extension contact have better access to farming information and professional assistance on farming activities to increase production and productivity in the sector (Abeje et al. 2019; Kebede et al. 2014). However, other studies revealed that an increase in the frequency of visits by development agents positively impacted livelihood diversification strategies (Bealu 2019; Gautam and Andersen 2016; Oduniyi and Tekana 2019). Monika et al. (2017), a study conducted in India, also established that farmers who attend farming training regularly are more likely to diversify their cropping systems. A summary of the key factors of social capital influencing diversification is presented in Table 6.

**Table 6:** Summary of key findings of social capital reported in reviewed studies

Main findings	Factors	Influence on diversification	Reference
Membership of households in any developmental group or organization and access to extension services can determine the adoption of livelihood diversification strategies.	Membership in development group or farmer organization	+	Abeje et al. (2019); Bealu (2019); Cynthia (2018); Dilruba and Roy (2012); Gautam and Andersen (2016); Shanta et al. (2018)
		-	Akaakohol and Aye (2014); Kebede et al. (2014);Alemayehu et al. (2021)
	Access to Agricultural Extension Office or any relevant govt. institution	+	(Bealu 2019); Gautam and Andersen (2016); Monika et al. (2017); Oduniyi and Tekana (2019); Alemayehu et al. (2021)
		-	Abeje et al. (2019); Kebede et al. (2014)

### Contribution of livelihood diversification strategies

This section provides a detailed synthesis of the identified literature on the impact of livelihood diversification strategies in reducing poverty (related to SDG-1 “no poverty”) in relation to other associated SDGs (Goal 2: zero hunger, Goal 5: gender equality, Goal 8: decent work and economic growth, Goal 10: reduce inequalities, Goal 12: sustainable consumption/Goal 12: sustainable production, and Goal 13: climate action). In this study, we assessed the contribution of livelihood diversification strategies in reducing poverty from the perspective of its ability to increase smallholder’s income. Birthal et al. (2015) analyzed diversification under rain-fed region and found diversification in form of high-value crops strategy in India and discovered that marginal farmers who increase their area of high-value crop cultivation by 39% to 50% were able to escape from poverty. Thapa et al. (2018) conducted a study in Nepal and found that the households who adopted diversified livelihood strategies on their farms had a mean monthly per capita expenditure 28% higher than non-adopters with a lower headcount poverty ratio of 9%. Similarly, Mukherjee (2015) found that the aggregate net earnings were higher for those whose farms were diversified than those whose fields were adopting traditional farming systems in India. Michler and Josephson (2017) concluded that livelihood diversification strategies positively impact rural income with the potential to reduce rural household poverty in Ethiopia. Megbowon and Mushunje (2018) observed that agricultural diversification could reduce poverty by 12.7% for rural households in South Africa. Overall, the literature indicates that an increase in the number of livelihood activities would increase the income of the households by improving their purchasing power and overall family welfare (Bird and Shepherd 2003; Ellis and Mdoe 2003; Olaleye 2016). It implies that the households who can engage in diversified livelihood strategies have a lower likelihood of being poor. A summary of livelihood diversifications’ contribution to poverty reduction is presented in Table 7.

**Table 7:** Contribution of livelihood diversification

SGD Goal	Achievement	Benefit	Source
Goal 2: zero hunger Goal 12: sustainable consumption	Food security and nutrition	Diversification brings diversify households’ food and diets Producing vegetables and fruits is helpful for food security and eventually anemia status of individuals (particularly for pregnant women) Agricultural diversifications bring direct impact on food security and availability	Adem et al. (2018); Adjimoti and Kwadzo (2018); Barrett et al. (2001); Bealu (2019); Cynthia (2018); Ecker (2018); Fred and Daniel (2011); Gani et al. (2019); Jones et al. (2014); Mango et al. (2018); Meena (2018); Michael (2015); Sarah (2019); Waha et al. (2018); Zeba and Shazia (2016)
Goal 5: gender equality	Gender equality	Livelihood diversification can empower women	Joshi et al. (2003); Shanta et al. (2018); Habib et al. (2022a)
Goal 8: decent work and economic growth Goal 10: reduce inequalities	Increase in Income	Increases economic permanence Stability in agricultural income Raises choice of on-farm systems Stabilization and generation of employment as a result of an expanded on-farm season Crop diversification substantially increases income from farming activities	Adebola et al. (2018); Adem et al. (2018); Awudu and Anna (2001); Etea et al. (2019); Gautam and Andersen (2016); Gururaj et al. (2017); Raphael and Matin (2009); Stefan and Manfred (2005); Tanvir et al. (2015); Wouterse and Taylor (2008)
Goal 12: sustainable production	Sustainable crop production	Increases and stabilize agricultural production Decreases the risk occurring from cyclical causes Crop diversification could be good strategy for risk prevention due to sudden variations in prices of crop yield	M. H. Ahmed et al. (2017); U. I. Ahmed et al. (2017); Anjani et al. (2012); Benmehaia and Brabez (2016); Birthal et al. (2015); Burchfield and Poterie (2018); (Cynthia 2018); Ecker (2018); ("Gender Involvement in Rainfed Agriculture of Pothwar," 2007); Huang et al. (2014); Michler and Josephson (2017); Miltone (2015); Monika et al. (2017); Njeru (2013); Pomi et al. (2017); Habib et al. 2022b; Rahman (2009); Shanta et al. (2018)
Goal 13: climate action	Climate vulnerabilities	Enhances tolerance towards water-logging and drought Improves yield permanence Can serve up as insurance opposed to rainfall variability	Anjani et al. (2012); Dilruba and Roy (2012); Gautam and Andersen (2016); Huang et al. (2014); Habib et al. (2022a);Martin and Lorenzen (2016); Miltone (2015); Njeru (2013); Tanvir et al. (2015)

#### Food security and nutrition

Reducing food insecurity remains a significant public policy challenge in developing countries (Andualem and Ebrahim 2021). The assessment of a farmer’s livelihood diversification strategies as a factor of food security among small scale farmers has been of interest to agricultural researchers in these countries (Alemayehu et al. 2021). Food insecurity becomes severe in areas where households are highly dependent on undiversified livelihoods (Etea et al. 2019). According to the studies retrieved, the contribution of agricultural diversification to increased food security and nutrition in poor households is primarily positive (Geremew et al. 2017; Sarah 2015). Abeje et al. (2019) established, based on the analysis of food expenditures in Ethiopia, that the food security situation of households who were able to diversify their income stream was better than households that could not adopt livelihood diversification strategies. Michael (2015) found that according to the Global Food Security Index (GFSI), households in Nigeria practicing agricultural diversification were 63% food secure. Gani et al. (2019) revealed that households in Nigeria that adopted livelihood diversification strategies fell short of the recommended calorie intake by 20%, while those who did not adopt livelihood diversification fell short by 35%.

In a study conducted in Ethiopia, Etea et al. (2019) concluded that there was a positive relationship between diversification and food security. Their findings revealed that due to lower adoption of diversification strategies, a majority of the households were food insecure in the study area. Zeba and Shazia (2016) showed that the diversification of cropping patterns in India was considered one of the crucial means to minimize risk and overcome food insecurity. Similarly, Makate et al. (2016) revealed a positive and significant impact of diversification on crop productivity, food security, and nutritional indicators in Zimbabwe. Douxchamps et al. (2015) also showed a positive impact of diversification on food security in West Africa. The findings of these studies reveal that households were more food secure with livelihood diversification strategies than those undertaking subsistence farming. It shows that as the number of livelihood strategies increases, the food security situation improves in most cases for rural households (Adjimoti and Kwadzo 2018; Bealu 2019; Ecker 2018). The prevalence of food insecurity was high in areas with a low level of income diversification (Etea et al. 2019). In summary, there is a clear indication of a positive association between livelihood diversification and food security in developing countries.

#### Gender equality

A gender system approach adds an important and unknown aspect to the literature on gender and livelihood diversification in developing countries (Sarah 2015; Habib et al. 2022c). Men have been dominant in the undertaking of most livelihood strategies (Kebede et al. 2014; Shanta et al. 2018) because of higher access to cash (Alemayehu et al. 2021; Long and Joanna 2018; Mulia et al. 2021) and other profitable interventions in non-farm livelihood strategies (Shanta et al. 2018; Silvestri et al. 2015). This review found a limited number of studies investigating and establishing a relation between livelihood diversification and gender. The indirect impact of livelihood diversification on gender equality is still missing from the literature. As most research has focused on men and women’s determinants of livelihood diversification, none has gone beyond and explored the impact of diversification on gender equality in providing equal wage rates and educational and health services for both men and women in developing countries’ context.

A few key concepts can be drawn from the available literature. Some researchers argue that newly developed agricultural markets are becoming more supportive of females’ participation in the management of finance in a male-dominated society (Buhl and Homewood 2000), where men are usually the leading player in livelihood diversification activities and generally the recipient of the ensuing benefits (Franklin 2010). Hailemariam et al. (2013) observed that adopting agricultural diversification in Ethiopia significantly enhanced the average female labor demand and instructed that this may negatively affect larger households by diverting time from food preparation and childcare. Franklin (2010) conducted a study in Malawi. He found that a female-headed household in the study area had low agricultural income, discouraging women participation in livelihood diversification strategies. Kebede et al. (2014) also found a positive dimension of agricultural diversification on gender. They concluded that if there is an increase in the agricultural diversification system, there would be a significant increase in female labor demand. Still, he further warned that this increase in women labor does not guarantee that they would spend the extra money they earn because men usually decide on financial matters. Franklin (2010) noted that males and females have traditionally had separate roles and duties, a concept that cannot be changed overnight in the developing world. Shanta et al. (2018) revealed that women exposed to outdoor market activities faced health and security issues in Nepal. Overall, the studies did not provide a clear picture of gender equality after having livelihood diversification but only presented their role in livelihood diversification strategies.

#### Increase in income level

The review revealed that there is a growing body of literature on the impact of livelihood diversification on improved income levels (Adebola et al. 2018; Adem et al. 2018; M. H. Ahmed et al. 2017; Barrett et al. 2001; Etea et al. 2019; Raphael and Matin 2009; Shakila et al. 2019; Wouterse and Taylor 2008), with most studies revealing a positive impact. A significant positive association between livelihood diversification and household income was found by Makate et al. (2016) in Zimbabwe, by Adjimoti and Kwadzo (2018) in Benin, and by Perz (2005) in the Brazilian Amazon. Nyikahadzoi et al. (2012) estimated a 21% average increase in farm income of the entire sample in the analysis. In contrast, Thapa et al. (2018) found a strong positive relationship between livelihood diversification and income, with a 28% higher consumption pattern for the household who adopted diversification than those who did not adopt livelihood diversification strategies in Nepal. Makate et al. (2016) remarked that expanded production from diversified cropping systems (crop rotations, intercropping) stemmed in higher income for farmers in Zimbabwe. Because agricultural diversification by adopting diverse cropping systems tends to decrease the chances of crop failures, this further improves crop yields which leads towards high standard trade with an increase of household income level (Anjani et al. (2012). Huang et al. (2014) found that with the increase of agricultural diversification in China, households who were unable to find jobs are now enjoying a better standard of living with the increase of livelihood diversification strategies on their farms. Similarly, Sarah (2019) indicated that non-farm livelihood strategies reduce the employment limitations of agricultural seasons by permitting farmers to earn more regular income throughout the year while permitting the creative combination of farm and non-farm activities. Finally, Basu (2014) demonstrated that, in India, agroforestry, a diversified agricultural system, offered a better livelihood outcome for the poor communities through the provision of employment generation and economic and food security. Overall, this synthesis provides sufficient evidence that a positive association exists between livelihood diversification and increased income levels in developing countries.

#### Sustainable crop production

Diversification in agriculture provides an opportunity to regenerate and conserve land and enhance agricultural productivity. Huang et al. (2014) found that farmers diversify their crops to mitigate natural disasters’ risks and negative impacts. Miltone (2015) highlighted that increasing diversity within farming systems is essential in helping farmers deal with greater climate variability and sustain their crop yield. By adopting a diversified farming system that promotes ecosystem services for pest and disease control and resilience to climate change variability, the production system is more generally resilient and sustainable to environmental change (Joshi et al. 2003; Waha et al. 2018). It reduces risk and optimizes crop productivity (Burchfield and Poterie 2018). Shanta et al. (2018) observed that agricultural diversification could have tremendous impacts on agro socio-economic areas and sustain better cropping systems. Crop diversification is considered one of the most cost-effective ways of reducing uncertainties in farmers’ income, especially among poor smallholder farmers (Njeru 2013). Anjani et al. (2012) concluded the agricultural diversification for Indian farmers provide sustainable crop productivity and generate employment opportunities for the rural youth. This implies that livelihood diversification in farming sector is crucial to maintain and sustain agricultural growth for farm-based rural communities in developing countries.

#### Climate change vulnerabilities

Climate change represents a significant threat to the current rural livelihood system and poses severe challenges to poor smallholder farmers who live and earn in rural areas. Several studies (Abid et al. 2016; Gentle and Maraseni 2012; Mulwa et al. 2017) from selected literature evaluated climate change as a threat to rural livelihoods. Livelihood diversification is often considered an essential strategy for dealing with climate change vulnerabilities (Basu 2014; Habib et al. 2015; Imran et al. 2018). In the selected list of studies, the focus of some studies was to observe farmers’ response against climatic vulnerabilities and the influencing factors of these vulnerabilities in adopting livelihood diversification strategies on their farms (Zimmerer 2014). Distress diversification is where diversification is seen as a strategy of spreading risk to reduce vulnerability to unpredictable crises such as floods, droughts, illness, and the seasonal fluctuations of natural resources (Allison and Horemans 2006; Ellis 2000; Smith 2004). It was observed that smallholder famers were diversifying their farms to mitigate the adverse effects of climate change on their livelihoods (Basu 2014; Philip and Leslie 2014). Gentle and Maraseni (2012) also indicated that crop diversification was a wise strategy to minimize productivity loss for small farmers in the context of climatic shocks in Nepal. In India, areas associated with harsh climates were more likely to see the adoption of livelihood diversification strategies (Gururaj et al. 2017). Makate et al. (2016) concluded that smallholders in Africa who diversify their farms due to climatic risk were more secure in food, income crop production, and nutrition. In India, Dilruba and Roy (2012) determined that household decisions to diversify crops were significantly influenced by their experiences of extreme weather events in the previous year. Such results are understandable because farmers’ behaviors are usually based on their experiences and expectations. From the selected studies, we can conclude that there is a positive association between climate change vulnerabilities and livelihood diversification in developing countries as it is used as a mitigation strategy against natural disasters.

## Conclusion

This systematic review synthesizes how livelihood capitals influence livelihood diversification strategies adoption and the impact of adopted livelihood diversification strategies in reducing poverty (SDG-1 “no poverty”) in relation to other SGDs in a developing country context. This systematic review reveals that human and natural capitals are the significant factors influencing livelihood diversification strategies. The impact of livelihood diversification in reducing poverty is reflected through improved food security and nutrition conditions, sustainable crop production, increased income level, sustainable crop production, and better adaptation to climate vulnerabilities. This review also suggests that better access towards livelihood capitals plays a crucial role in adopting livelihood diversification strategies and is a pathway to achieving the SDG-1 “no poverty” objective. However, measuring the actual economic impact of livelihood diversification on SDG-1 “no poverty” is problematic because only a few studies have thoroughly analyzed the impact of livelihood strategies on poverty alleviation. A holistic evaluation of the different livelihood diversification strategies on SDG-1 “no poverty” could better inform policymakers about the real economic impacts of this strategy for future promotional programs or policies. This literature also revealed the scarcity of studies analyzing the relationship between livelihood diversification and gender. Therefore, future research should thoroughly analyze the influence and contribution of livelihood capitals on livelihood diversification strategies and the impact of these strategies on food security and nutrition, gender equality, and climate change vulnerabilities. In addition, policymakers should consider introducing developmental policies that could provide smallholder farmers (including women) with access to natural (land, water), financial (formal credit facilities), and physical (access to mobility services) to encourage their participation in livelihood diversification activities. The synthesis of the impacts of livelihood diversification strategies in improving SDG-1 “no poverty” provided here can increase awareness and reinforce efforts for more sustainable rural livelihood strategies in developing countries.

## Data Availability

The data sharing is not applicable to this article as no new data were created or analyzed in this study.
